# Witnesses Three

**Published:** 1863-03

**Authors:** 


### WITNESSES THREE.

Shortly before he died, Patrick Henry, laying his hand on the Bible, said:

"Here is a Book worth more than all others, yet it is my sad misfortune never to have read it, until lately, with proper attention."

With voice and gesture, pertinent, and all his own, John Randolph said:

"A terrible proof of our deep depravity is, that we can relish and remember anything better than "the Book."

When the shades of death were gathering around Sir Walter Scott, he said to the watcher, "Bring the Book."

"What book," asked Lockhart, his son-in-law.

"There is but one book," said the dying man.

With such testimony as to the value of the Sacred Scriptures, reiterated by the great and good, in all ages, it is a sealed book to many; it is voted to be excluded from our public schools, and multitudes of children are growing up ignorant of its histories, ignorant of its immortal truths, and profoundly unconscious that, to it and to its teachings, they owe all that is of solid worth in social life, in civil liberty, in human elevation, and in the hope of an immortal existence.

